# Metabolomics and transcriptomics unravel the mechanism of browning resistance in *Agaricus bisporus*

**DOI:** 10.1371/journal.pone.0255765

**Published:** 2022-03-16

**Authors:** Zhi-Xin Cai, Mei-Yuan Chen, Yuan-Ping Lu, Zhong-Jie Guo, Zhi-Heng Zeng, Jian-Hua Liao, Hui Zeng

**Affiliations:** Institute of Edible Mushroom, Fujian Academy of Agricultural Sciences, National-Local Joint Engineering Research Center for Breeding and Cultivation of Featured Edible Mushroom, Fuzhou, Fujian, China; ICAR-National Institute of Plant Biotechnology, New Delhi, INDIA

## Abstract

*Agaricus bisporus* is widely consumed on the world market. The easy browning of mushroom surface is one of the most intuitive factors affecting consumer purchase. A certain cognition on browning mechanism has been made after years of research. At present, people slow down the browning of mushrooms mainly by improving preservation methods. In addition, breeding is also a reliable way. In the production practice, we have identified some browning-resistant varieties, and we selected a browning-resistant variety to compare with an ordinary variety to reveal the resistance mechanism. Using transcriptomics and metabolomics, the differences in gene expression and metabolite levels were revealed, respectively. The results showed that differentially expressed genes (DEGs) like *AbPPO4*, *AbPPO3* and *AbPPO2* were differently expressed and these DEGs were involved in many pathways related to browning. The expression of *AbPPO* expression play an important role in the browning of *A*. *bisporus* and multiple PPO family members are involved in the regulation of browning. However, the resistance to browning cannot be judged only by the expression level of *AbPPOs*. For metabolomics, most of the different metabolites were organic acids. These organic acids had a higher level in anti-browning (BT) than easy-browning varieties (BS), although the profile was very heterogeneous. On the contrary, the content of trehalose in BS was significantly higher than that in BT. Higher organic acids decreased pH and further inhibited PPO activity. In addition, the BS had a higher content of trehalose, which might play roles in maintaining PPO activity. The difference of browning resistance between BS and BT is mainly due to the differential regulation mechanism of PPO.

## Introduction

*Agaricus bisporus* (*A*. *bisporus*) is a widely cultivated edible mushroom with high yields and enormous consumption. *A*. *bisporus* has high nutritional value with low calories and high protein. The activation of flavour amino acids and nucleotides, such as soluble sugar, polyols, free amino acids, 5’-nucleotides and sodium glutamate (MSG), in *A*. *bisporus* makes it a unique aroma and flavor [1, 2]. In addition to its nutritional value, *A*. *bisporus* is widely favoured because of its numerous medicinal values. *A*. *bisporus* contains tyrosinase, rich selenium and vitamins, significantly reducing cholesterol and blood pressure [3]. In recent years, due to the successful realization of mushroom’s deep culture technology, people can use mushroom mycelium to produce some substances such as protein, mycosaccharide and oxalic acid. In addition, *A*. *bisporus* is rich in unsaturated fatty acids, reducing liver cirrhosis, arteriosclerosis, obesity, heart disease and so on [4]. However, mushroom preservation has always been a complex problem in the industry, and browning is challenging to overcome.

Browning is one of the important factors affecting the quality of edible fungi, which seriously affects the quality and nutritional value of edible fungi. The browning factors include internal factors (e.g., variety, moisture content, maturity, respiration rate, microorganism) and external factors (e.g., temperature, humidity, gas composition, mechanical damage, etc.). The cause of browning can be divided into two categories: enzymatic and non-enzymatic browning. Enzymatic browning is considered to be the leading cause of postharvest browning of edible fungi. Previous studies reported that the main enzymes causing browning in edible fungi are tyrosinase, such as *A*. *bisporus*, White Flammulina, and Coprinus [5–7]. Tyrosinase catalyzed the synthesis of L-dopa from phenolic substrates, followed by the further synthesis of dopaquinone, and finally formed melanin [8]. However, tyrosinase catalyzed phenolic substrates are different in different edible fungi. In addition, microbial infection is also the cause of browning of *A*. *bisporus*, such as *Pseudomonas tolaasii*, (*P*. *tolaasii*). The non-enzymatic browning mainly refers to the color deepening of mushroom surface caused by oxidation in contact with air, which might lead to the destruction of nutrients and quality deterioration. Li et al determined the color difference, total phenol content and enzyme activity of *A*. *bisporus* during storage and suggested that the polyphenol oxidase (PPO) and phenylalanine-ammonia-lyase (PAL) in *A*. *bisporus* are the main factors causing browning [5].

In the present study, we collected several *A*. *bisporus*, including varieties with browning susceptibility, varieties with browning resistance and varieties with intermediate traits. We used transcriptional and metabolomics to analyze the browning mechanism of *A*. *bisporus*. We hypothesized that browning is due to the interaction of certain genes and specific metabolites. This study will provide a theoretical basis for *A*. *bisporus* browning.

## Materials and methods

### Samples preparation

Fruitbodies of mushroom (*A*. *bisporus*), each with the cap arithmetic mean diameter of 30–45 mm at commercial mature stage, were harvested from local climate experiment mushroom room of the Institute of Edible Mushroom, Fujian Academy of Agricultural Sciences of China, and transported under ambient conditions within 5 minutes to the laboratory. *A*. *bisporus* strains were grown on Petri dishes (90 mm Ø) containing 20 ml of the following culture media: malt extract agar (MEA, Bioxon: 20 g·l-1 malt extract, 15 g·l-1 agar). Culture media were sterilized at 121°C for 20 min. Each Petri dish was inoculated in the center with a 0.5 cm diameter disc of previously uniformly colonized MEA of one strain and incubated at 22, 25 and 28°C, in the dark. From four batches of mushroom, containing 80 fruits each, 200 samples were selected on the basis of the uniformity of maturity and appearance so that any open cap, nonwhite surface color, mechanical damaged, and defected mushrooms were excluded. In order to identify the browning sensitivity of mushrooms from different fungal sources, they were randomly divided into 3 groups (40 in each group) and stored at standard temperature and relative humidity (3±1°C and 92±2% R.H.). The fresh fruiting bodies were collected and the stipe was removed. The caps were evenly scratched with a sandpaper and stand for 10 minutes. The whiteness value of the caps surface was measured with a whiteness meter (Qtboke YQ-Z-488, Hangzhou, China) [9]. All detection were repeated three times with at least six biological and five technical replicates per treatment.

### Global gene transcriptional analysis

According to the results of browning sensitivity identification, the anti-browning (BT) and easy-browning varieties (BS) were selected for transcriptome sequencing. Two samples were randomly selected as biological repeats in each group. To construct each cDNA library, total cellular RNA was extracted from fibroblasts using Trizol reagent (Invitrogen, CA, USA). Each cDNA library was constructed according to the manufacturer’s guidelines, and next-generation sequencing was carried out in the company (Allwegene Co. Ltd, Beijing, China), using an Illumina HiSeq 4000 platform (pair-end 150bp).

For sequence data analysis, the Trimmomatic package (version 0.32) was used to obtain clean reads under default parameters. Then, the reference genome sequence of *A*. *bisporus* was downloaded from fungi ensemble database (http://fungi.ensembl.org/Agaricus_bisporus_var_bisporus_h97/Info/Index). All the clean reads were mapped to the reference using Tophat2 (version 2.1.1) with default parameters. After the number of reads mapped to each gene was counted, the FPKM (fragments per kilobase per million fragments) method was used for normalization through (RNA-Seq by Expectation Maximization) RSEM (https://deweylab.github.io/RSEM/), and the lowly expressed genes (FPKM < 1) were filtered in each sample [10]. DESeq2 was employed to calculate the log 2-fold change (log2FC) and probability value for each gene in every comparison, and strict criterion were used (log2FC > 2 or log2FC < −2, false discovery rate < 0.01). To analyze the potential functions of the DEGs, we enriched the DEGs according to Gene Ontology (GO) and Kyoto Encyclopedia of Genes and Genomes (KEGG) database with KOBAS (version 2.0) software. The threshold of significance was defined as FDR < 0.05.

### Sample preparation and analysis by UPLC-QTOFMS

In order to identify the difference of metabolites between the two varieties of *A*. *bisporus*, we selected 12 samples (six in each group, two groups) for metabolomics analysis. The untargeted metabolomics was carried out by ultra-performance liquid chromatography-quadrapole time of flight mass spectrometry (UPLC-QTOFMS) according to the previously established methods [11–13]. The freeze-dried samples were crushed using a mixer mill (MM 400, Retsch) with a zirconia bead for 1.5 min at 30 Hz. Then 100 mg powder was weighed and extracted overnight at 4°C with 1.0 ml 70% aqueous methanol containing 0.1 mg/L lidocaine for internal standard. Following centrifugation at 10000 g for 10min, the supernatant was analyzed using an LC-ESI-MS/MS system. Briefly, 2 μl of samples were injected onto a Waters ACQUITY UPLC HSS T3 C18 column (2.1 mm*100 mm, 1.8 μm) operating at 40°C and a flow rate of 0.4 mL/min. The mobile phases used were acidified water (0.04% acetic acid) (Phase A) and acidified acetonitrile (0.04% acetic acid) (Phase B). Compounds were separated using the following gradient: 95:5 Phase A/Phase B at 0 min; 5:95 Phase A/Phase B at 11.0 min; 5:95 Phase A/Phase B at 12.0 min; 95:5 Phase A/Phase B at 12.1 min; 95:5 Phase A/Phase B at 15.0 min. The effluent was connected to an the Synapt G2-S QTOFMS system (Waters, Milford, MA).

### Metabolomic data analysis

To produce a matrix containing fewer biased and redundant data, peaks were filtered to remove the redundant signals caused by different isotopes, in-source fragmentation, K+, Na+, and NH4+ adduct, and dimerization. According to the detected peaks, metabolites were identified by searching internal database and public databases [MassBank (https://massbank.eu/MassBank/), KNApSAcK (www.knapsackfamily.com/KNApSAcK/), HMDB (https://hmdb.ca/), MoTo DB [14], and METLIN [15]). Orthogonal projection to latent structures-discriminant analysis (OPLS-DA) was performed for classification and discriminant analysis of the samples. OPLS-DA was applied in comparison groups using R software according to a previous report [16]. A variable importance in projection (VIP) score of (O)PLS model was applied to rank the metabolites that best distinguished between two groups. The threshold of VIP was set to 1. In addition, T-test was also used as a univariate analysis for screening differential metabolites. Those with a P value of T test <0.05 and VIP ≥ 1 were considered differential metabolites between two groups. Then, metabolites were mapped to KEGG metabolic pathways for pathway analysis and enrichment analysis.

### Statistical analysis

Data are presented as the mean ± S.D. Statistical analyses were performed using IBM SPSS 20 (IBM, Armonk, NY). Comparison between groups was performed using one-way analysis of variance, followed by the Scheffe’s procedure for the post hoc test. P < 0.05 was considered to be statistically significant.

## Results

### Evaluation of browning resistance

**Fig 1:**
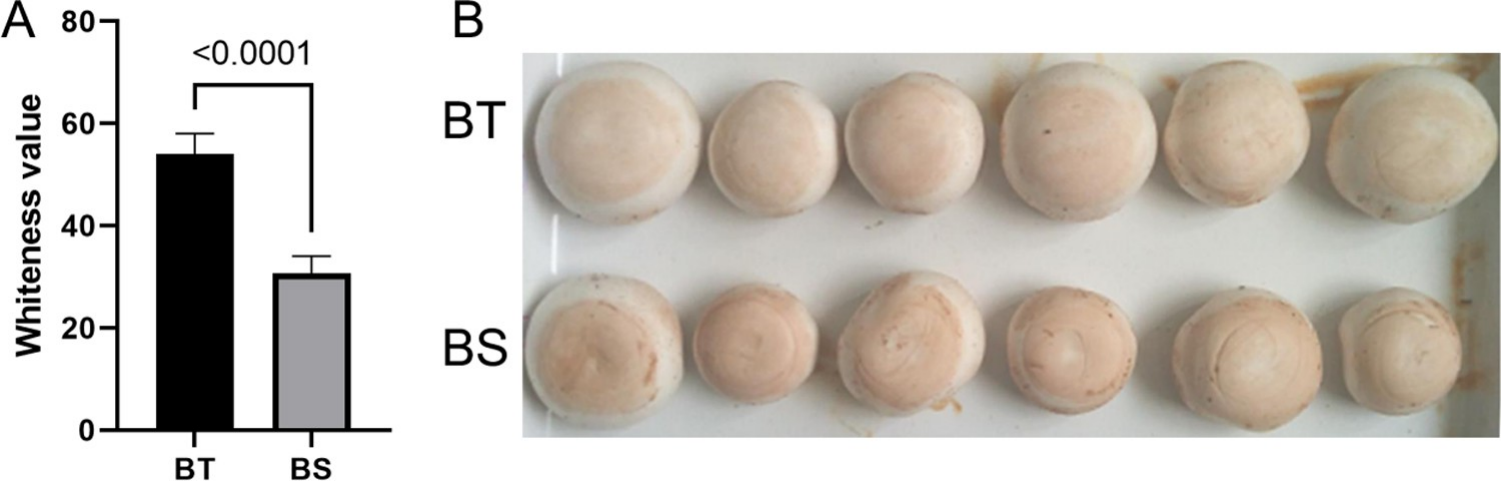
Evaluation of browning resistance. BT notes the anti-browning mushroom variety; BS notes the easy-browning variety. A shows the statistical results of the whiteness values of the two varieties of mushrooms, using the T-test. B shows the intuitive difference between the two varieties. BT: anti-browning mushroom. BS: easy-browning mushroom.

The resistance identification of two varieties of BT and BS respectively, we found that BT has significantly stronger browning resistance than BS (**Fig 1A**). The whiteness value of BT exceeds 80% of the whiteness value of BS (**Fig 1B**).

### Summary of transcriptome data

A total of 9,505 genes were detected in 4 samples. Percentages of reads mapped to the genome ranged from 69.27%–73.89%. After removing adapters, low-quality reads and contaminant rRNA reads, the remaining high-quality RNA-seq datasets contained 6.0–7.2 Gb of clean data from each sample (**S1 Table**). The Q20, Q30 and GC content values were 96.81–97.14%, 91.87–92.46% and 49.22–49.50%, respectively (**S1 Table**). Totally,1,133 DEGs were identified in the four samples, including 522 up-regulated and 611 down-regulated DEGs (BT *vs*. BS).

### Enrichment of functional genes

**Fig 2:**
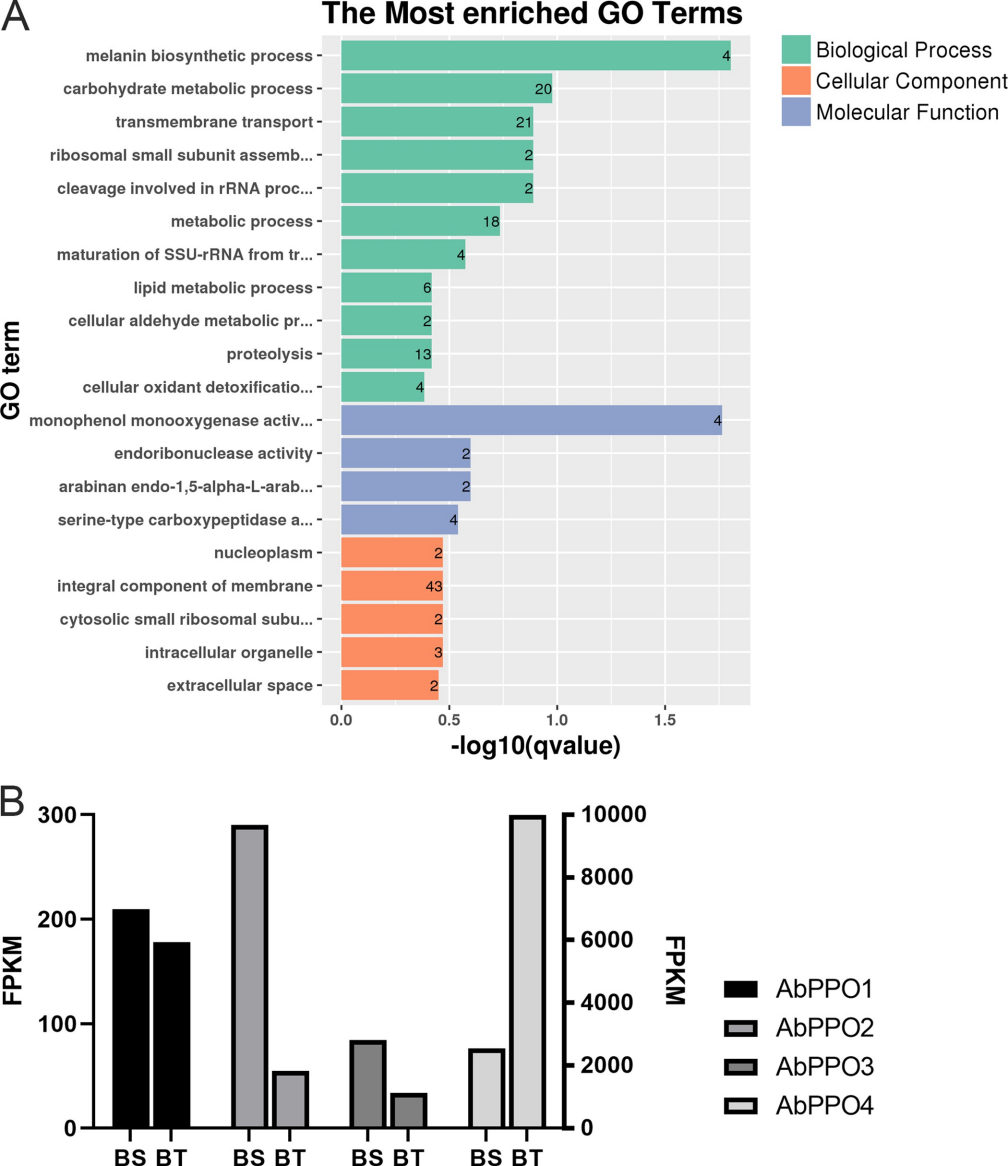
GO functional classification results. BT notes the anti-browning mushroom variety; BS notes the easy-browning variety. A shows the GO enrichment results. The left side indicates the GO terms name. The number on the right bar indicates the number of genes. B shows the expression level of *AbPPO* gene family members. The left ordinate represents the expression level of *AbPPO1*, *AbPPO2* and *AbPPO3*. The right ordinate represents the expression level of *AbPPO4*. BT: anti-browning mushroom. BS: easy-browning mushroom.

To determine the main biological functions filled by BT and BS DEGs, these transcripts were firstly mapped to GO terms in BLAST2GO analysis. The enrichment results showed that oxidation-reduction process (GO:0055114) were significantly enriched and DEGs like *AbPPO4* (upregulated), *AbPPO3* (downregulated) and *AbPPO2* (downregulated) were differently expressed. Also, melanin biosynthetic process (GO:0007093) were significantly enriched (**Fig 2A**). Interestingly, all the DEGs mapped to melanin biosynthetic process term were polyphenol oxidase members, including *AbPPO1* to *AbPPO4* and three of them were differently expressed (**Fig 2B**). The results of GO enrichment indicated that *AbPPO* gene family played a key role in mushroom browning.

**Fig 3:**
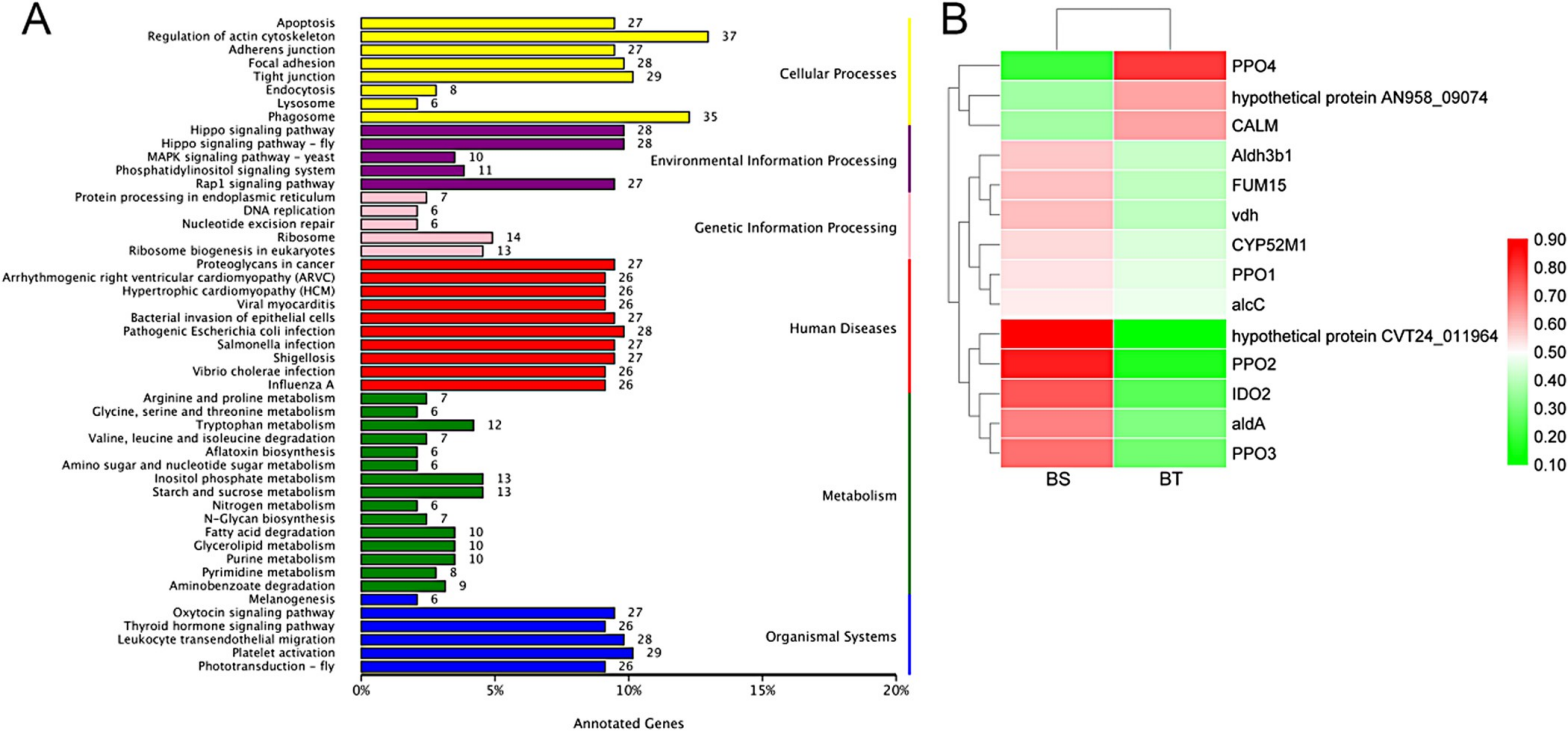
KEGG functional classification results. BT notes the anti-browning mushroom variety; BS notes the easy-browning variety. A shows the KEGG enrichment results. The left side indicates the KEGG pathways name. The number on the right bar indicates the number of genes. B shows the expression heatmap of candidate genes that obtained from the KEGG analysis. The red box represents the level of expression of candidate genes and the redder the color, the higher the expression level. BT: anti-browning mushroom. BS: easy-browning mushroom.

Then, we performed the KEGG enrichment analysis to determine the pathways involved in DEGs. Among all the 40 significantly enriched pathways, melanogenesis (ko04916), tryptophan metabolism (ko00380), tyrosine metabolism (ko00350), and taurine and hypotaurine metabolism (ko00430) might be related to the browning of mushroom, especially the melanogenesis pathway (**Fig 3A**). Then, the expression levels of all the DEGs in these four pathways were shown in a heatmap (**Fig 3B**). We found that *PPO* family members participate in the production of melanin. In addition, AldA, CALM, and two hypothetical proteins (hypothetical protein AN958_09074 and hypothetical protein CVT24_011964) genes were differently expressed between BS and BT.

### Summary of metabolomic data

**Fig 4:**
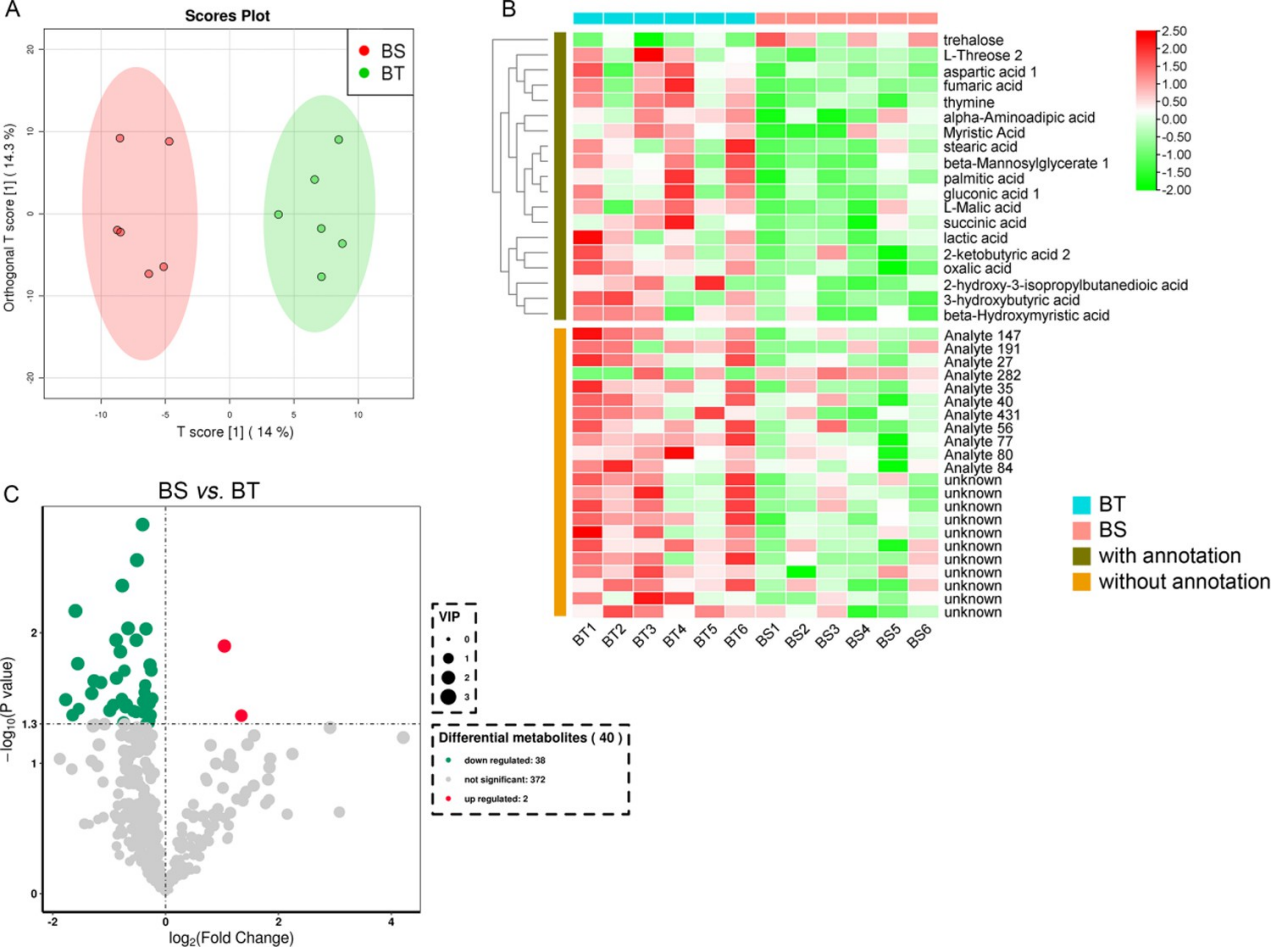
Metabolomics and differential metabolites. BT notes the anti-browning mushroom variety; BS notes the easy-browning variety. A shows the PCA results of all samples. B shows the heatmap of differential metabolites, the redder the color, the higher level of metabolites. C shows the volcano plot of differential metabolites. BT: anti-browning mushroom. BS: easy-browning mushroom.

The metabolomics data from the present LCMS-based comparative untargeted metabolomics study were highly reproducible. OPLS-DA score plots also clearly show that there were global metabolic differences between the two groups (**Fig 4A**). Totally, we identified 474 metabolites. There were significant differences in the contents of 40 metabolites between BS and BT (including 22 metabolites without valid annotation information, **Fig 4B and 4C**).

### Differential metabolites and pathways

By annotating on all the differential metabolism, we found that most of the different metabolites were organic acids, such as succinic acid, L-Malic acid, fumaric acid, etc. These organic acids had a higher level in BT than that in BS, although the profile was very heterogeneous. On the contrary, the content of trehalose in BS was significantly higher than that in BT. In order to further analyze the function of these metabolites, we used KEGG clustering to analyze the known metabolites. We found that 15 KEGG pathways were significantly enriched, including fatty acid biosynthesis (ath00061), tyrosine metabolism (ath00350), citrate cycle (ath00020), etc (**Table 1**).

**Table 1:** KEGG enrichment results of different metabolites.

Pathway	Hits metabolisms
Citrate cycle (TCA cycle)	Succinic acid;L-Malic acid;Fumaric acid
Fatty acid biosynthesis	Stearic acid;Myristic acid;Palmitic acid
Glyoxylate and dicarboxylate metabolism	L-Malic acid;Succinic acid
Tyrosine metabolism	Fumaric acid;Succinic acid
Pyruvate metabolism	L-Malic acid;L-Lactic acid
Alanine, aspartate and glutamate metabolism	Fumaric acid;Succinic acid
Biosynthesis of unsaturated fatty acids	Palmitic acid;Stearic acid
Fatty acid elongation in mitochondria	Palmitic acid
Propanoate metabolism	Succinic acid
Butanoate metabolism	Succinic acid
Carbon fixation in photosynthetic organisms	L-Malic acid
Glycolysis or Gluconeogenesis	L-Lactic acid
Starch and sucrose metabolism	Trehalose
Fatty acid metabolism	Palmitic acid
Arginine and proline metabolism	Fumaric acid

## Discussion

In our mushroom breeding practice, we found that most *A*. *bisporus* are prone to browning, while some are resistant to browning. In order to screen the differences between the two mushroom varieties, we analyzed the transcriptome and metabolomics data of the fruiting body of the mushroom.

**Fig 5:**
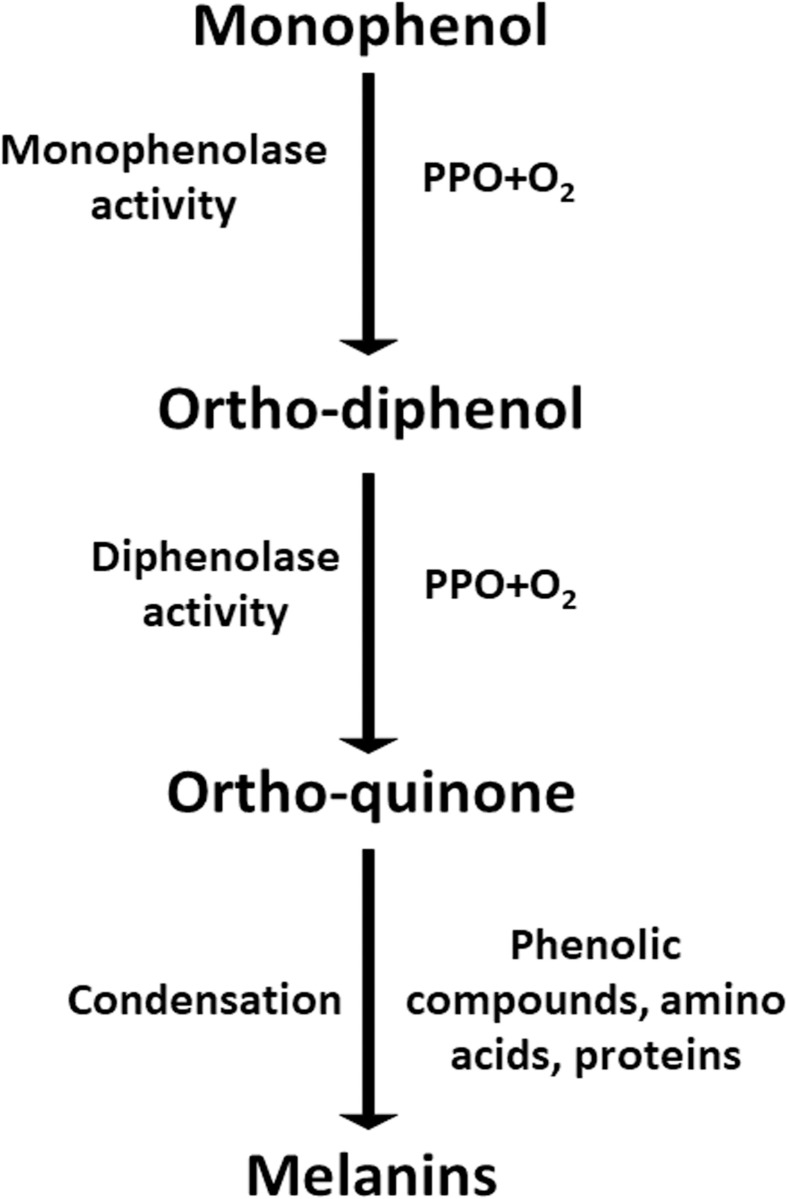
Schematic figure of browning process. PPO, polyphenol oxidases.

The transcriptome analysis showed that the *PPO* family members had the greatest effect on *A*. *bisporus* browning. Polyphenol oxidases are implicated in various biological functions in diverse systems (**Fig 5**). In addition to a role in black/brown pigment biosynthesis, PPOs may also have protective roles in plants against pathogens and environmental stress [17]. *PPO* family members participate in multiple biological pathways, and some of them proved to defense against diverse diseases and biotic stress in plants [18, 19]. As we know, in the presence of oxygen, the PPO enzyme changes substances known as phenolic compounds (through a process of oxidation) into different compounds called quinones. The quinones then react with other compounds to form melanin, then the fruit and vegetables turn brown [20, 21].

The present results identified four *AbPPO* gene family members (*AbPPO1*, *AbPPO2*, *AbPPO3* and *AbPPO4*) differentially expressed between the two groups, three (*AbPPO1*, *AbPPO2*, *AbPPO3*) of which were highly expressed in BS but lower in BT. The expression of *AbPPO4* is special, whose absolute expression level was the highest of all the identified *PPO* members, and *AbPPO4* had a significantly higher expression level in BT than that in BS. These results suggest that *AbPPO1*, *AbPPO2*, *AbPPO3* and *AbPPO4* play different roles in mushroom browning, and that individual PPO gene family members are under elaborate controls to differentially express in response to specific developmental and environmental cues. According to the results of this study, higher expression of *AbPPO4* may play a positive role in the anti-browning effect of *A*. *bisporus*. Lei et al reported that that *AbPPO3* and *AbPPO4* contributed to the browning of mushrooms, however, the expression of *AbPPO* genes were significantly different in different parts [22]. Also, Lei found that the *AbPPO* expression trend and browning trend were different in different tissues of mushrooms [22]. Hence, we speculated that the browning of *A*. *bisporus* is related to *AbPPO* expression, but the resistance to browning cannot be judged only by the expression level of *AbPPOs*.

Metabolomics analysis has good performance on screening the different compounds in mushroom samples. In the present study, many organic acids (such as lactic acid, malic acid, and fumaric acid) had a higher level in BT than BS. Liu et al reported that as the concentration of citric acid increased, the activity of PPO decreased gradually [23]. Zhou reported that malic acid, citric acid and buffer with the same pH had a similar effect on the relative activity of PPO, indicating the inhibition of PPO induced by malic acid and citric acid might be mainly attributed to the decrease of pH [24]. Previous studies held that acidulants could change the pH of the peripheral environment of PPO, which further induces the decrease of activity [23, 25]. Besides, organic acids inhibited PPO through chelation of the copper were reported. Yoruk and Marshall found that oxalic acid chelated the copper and diminished the catechol-quinone product formation. Kojic acid was reported to inhibit PPO by forming a strong chelate with copper [26]. In this present study, L-Malic acid, succinic acid, fumaric acid, aspartic acid and palmitic acid had a higher level of organic acid. These organic acids maintained a low pH value, which inhibited the PPO activity. Additionally, the content of trehalose in all metabolites is very high, and there is a great difference between the two groups, which makes us curious about the role of trehalose in mushroom browning. Trehalose has a higher level in BS than BT. It has been shown that trehalose can protect proteins and cellular membranes from inactivation or denaturation caused by various stress conditions, including desiccation, dehydration, heat, cold, and oxidation [27]. The higher content of trehalose in BS might maintain a high activity of PPO, which leads to the browning of mushroom; however, this is only our conjecture, and more experiments are needed to confirm it.

## Conclusion

The expression of *AbPPO* expression play an important role in the browning of *A*. *bisporus* and multiple PPO family members are involved in the regulation of browning. However, the resistance to browning cannot be judged only by the expression level of *AbPPOs*. In BT, higher organic acids decreased pH and further inhibited PPO activity. In addition, the BS had a higher content of trehalose, which might play roles in maintain the activity of PPO. Therefore, the difference of browning resistance between BS and BT is mainly due to the differential regulation mechanism of PPO.

## Supporting information

S1 Table Summary of transcriptome data. (DOCX)
