# Supplementary material for: Metabolomics and transcriptomics unravel the mechanism of browning resistance in Agaricus bisporus
Source: PLoS One. 2022 Mar 16;17(3):e0255765. doi: 10.1371/journal.pone.0255765 (PMC8926301; doi:10.1371/journal.pone.0255765)
Supplement: S1 Table — (DOCX) [file pone.0255765.s001.docx]

**S1 Table Summary of transcriptome data**

| Sample ID | Read Sum | Base Sum | Q20(%) | Q30(%) | GC(%) |
| --- | --- | --- | --- | --- | --- |
| BS_r1 | 24,070,648 | 7,221,194,400 | 96.94% | 92.14% | 49.39% |
| BS_r2 | 22,101,726 | 6,630,517,800 | 97.14% | 92.46% | 49.22% |
| BT_r1 | 20,196,242 | 6,058,872,600 | 96.91% | 92.03% | 49.50% |
| BT_r2 | 20,445,011 | 6,133,503,300 | 96.81% | 91.87% | 49.34% |

BT notes the anti-browning mushroom variety; BS notes the easy-browning variety. R1 and r2 means the biological replicates.
